# Predictors of post-stroke fever and infections: a systematic review and meta-analysis

**DOI:** 10.1186/s12883-018-1046-z

**Published:** 2018-04-23

**Authors:** Maja Wästfelt, Yang Cao, Jakob O. Ström

**Affiliations:** 1 Department of Neurology, School of Medical Sciences, Örebro University, Örebro, Sweden; 2 Clinical Epidemiology and Biostatistics, School of Medical Sciences, Örebro University, 70182 Örebro, Sweden; 3 Unit of Biostatistics, Institute of Environmental Medicine, Karolinska Institutet, 17177 Stockholm, Sweden; 4 Department of Clinical Chemistry, Institution of Clinical and Experimental Medicine, Linköping University, Linköping, Sweden

## Abstract

**Background:**

Fever after stroke is common, and often caused by infections. In the current study, we aimed to test the hypothesis that pneumonia, urinary tract infection and all-cause fever (thought to include at least some proportion of endogenous fever) have different predicting factors, since they differ regarding etiology.

**Methods:**

PubMed was searched systematically for articles describing predictors for post-stroke pneumonia, urinary tract infection and all-cause fever. A total of 5294 articles were manually assessed; first by title, then by abstract and finally by full text. Data was extracted from each study, and for variables reported in 3 or more articles, a meta-analysis was performed using a random effects model.

**Results:**

Fifty-nine articles met the inclusion criteria. It was found that post-stroke pneumonia is predicted by age OR 1.07 (1.04–1.11), male sex OR 1.42 (1.17–1.74), National Institutes of Health Stroke Scale (NIHSS) OR 1.07 (1.05–1.09), dysphagia OR 3.53 (2.69–4.64), nasogastric tube OR 5.29 (3.01–9.32), diabetes OR 1.15 (1.08–1.23), mechanical ventilation OR 4.65 (2.50–8.65), smoking OR 1.16 (1.08–1.26), Chronic Obstructive Pulmonary Disease (COPD) OR 4.48 (1.82–11.00) and atrial fibrillation OR 1.37 (1.22–1.55). An opposite relation to sex may exist for UTI, which seems to be more common in women.

**Conclusions:**

The lack of studies simultaneously studying a wide range of predictors for UTI or all-cause fever calls for future research in this area. The importance of new research would be to improve our understanding of fever complications to facilitate greater vigilance, monitoring, prevention, diagnosis and treatment.

## Background

Infections after stroke are common, and the prevalence has been reported to be as high as 30%; one third consisting of pneumonia and another third of urinary tract infections (UTI) [1]. These infections are associated with higher morbidity and mortality [2]. However, fever after stroke can also be endogenous, commonly referred to as “central fever”, caused by immune system activation or effects of the brain lesion on thermoregulatory centers, and such episodes are often difficult to distinguish from infections [3]. Central fever has not been very well characterized, but is probably resistant to antibiotic treatment and antipyretic treatment and probably appears early after stroke [3]. Fever without an identified infection has been reported to occur in 14.8% of stroke patients [3], but this number is uncertain, and reasonably depends on how thoroughly the patients have been investigated for focal signs of infections. Regardless of the causes, elevated body temperature after stroke is associated with poor prognosis [4, 5].

By understanding the risk factors of different types of infections and fever, risk profiles of specific patients could be assessed, in turn facilitating the diagnostics in stroke patients with increased body temperature. A meta-analysis comparing risk factors for different fever-related complications is lacking. In this study it was hypothesized that pneumonia, urinary tract infection (UTI) and all-cause fever have different predictors, since they differ regarding etiology. Therefore the aim of this study was to perform a meta-analysis to compare the predictors for post-stroke pneumonia, UTI and all-cause fever.

## Methods

### Literature search

PubMed was searched through its inception to September 16th, 2016 by using the MeSH terms “Cerebral Infarction”, “Stroke”, “Cerebral Hemorrhage” and the free text terms “fever”, “infection”, “pneumonia” and “urinary tract infection”. The search was complemented by a free text search using the same terms and filtered for “Ahead of print” to catch studies which were not found in the MeSH term search. Two more searches was performed; 1) using MeSH terms “Stroke/complications”, “Stroke/epidemiology”, “Stroke/physiology”, “Stroke/prevention and control”, “Stroke/statistics and numerical data” and “Infection”; and 2) using MeSH terms “Stroke/complications”, “Stroke/statistics and numerical data” and “fever”. These searches were undertaken as a preliminary investigation before the main search was performed. However, these searches identified only one article not found by the main search described above, and hence this article was added to the systematic search results. We followed the standard criteria PRISMA (Preferred Reporting Items for Systematic Reviews and Meta-Analysis) [6] and MOOSE (Meta-analysis of Observational Studies in Epidemiology) [7] throughout the study.

### Selection criteria

These searches resulted in a total of 5294 articles, which were assessed manually first by title, then by abstract and finally by full text. Only studies in English was considered in the process. Grey literature was not included in the process. A selection was made based on the following inclusion criteria, aiming to include articles regardless of study design:

- Studies of patients with ischemic and/or hemorrhagic stroke.
- Studies performing multiple logistic regression analysis with the dependent variable pneumonia, UTI or fever (any definition), presenting the results in odds ratio (OR).

Studies including only patients with subarachnoid hemorrhage were excluded. Studies where all patients were treated at intensive care unit or in ventilator were also excluded from the sample.

### Data extraction

Author, publication year, study design, inclusion and exclusion criteria, number of included patients, definitions of outcome measures, study time period and covariates included in the regression model were extracted from all included studies by one investigator. In addition, all available results on predictors of pneumonia, UTI and all-cause fever were extracted.

Certain predictors were described differently in different articles, and categorized according to the following:

- Mechanical ventilation - Including tracheostomy, endotracheal intubation and endotracheal incision.
- Dysphagia - Including penetration of liquid on fiber endoscopic evaluation, abnormal bedside swallowing test during admission.
- Nasogastric tube - Including enteral feeding during admission.
- Hypertension - Including history of hypertension and hypertension diagnosis.

### Quality assessment

Included articles were assessed for risk of bias using a form from the Swedish Agency for Health Technology Assessment and Assessment of Social Services [8]. Each study was assessed according to the following fields: Selection bias (A1), Treatment and measurements (A2), Detection bias (A3), Attrition bias (A4), Reporting bias (A5), Conflict of interests (A6).

All fields were assessed and classified as one of the following: low risk of bias, average risk of bias or high risk of bias. In field A2 each article was considered with respect to the measurements of pneumonia, UTI and all-cause fever. Other outcome measures were not taken into account in the quality assessment.

From this assessment, the studies were categorized into three groups regarding quality by following criteria:

- Low risk of bias –one field to be assessed as average risk
- Average risk of bias – up to 3 fields assessed as average risk
- High risk of bias – 4 or more fields assessed as average risk or one field assessed as high risk

The quality assessment was only performed to shed light on the quality of the studies included, and was not used for exclusion of articles.

#### Statistical analysis

Specific predictors for an outcome were synthesized if at least three articles contributed to the data. The data was analyzed using a random effect model. The overall combined OR with 95% confidence interval (CI) and *p*-value were calculated. A predictor with a 95% CI not including 1 and *p* < 0.05 was considered statistically significant. I^2^ in percentage with *p*-values were calculated to describe heterogeneity among studies, I^2^ > 30% was considered a moderate to high heterogeneity. All the analyses were performed in STATA®14 software (StataCorp LLC, College Station, Texas) [9].

## Results

**Fig. 1:**
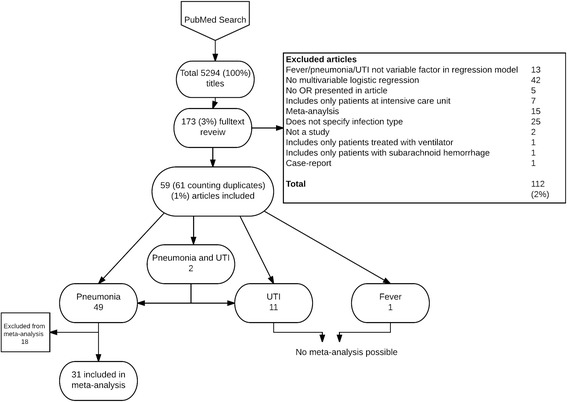
Screening and selection of articles. 2 articles were included in both pneumonia and UTI group, counting the duplicates the total sum adds to 61

In total, 5294 titles were identified by the searches. After manual screening, 174 articles were left for full text review. After reading the articles in full text, 111 articles were excluded leaving a total of 59 articles to be included in the sample for analysis (Fig. 1). Of these, 47 studied pneumonia, 9 studied UTI, 2 studied both pneumonia and UTI and 1 studied all-cause fever.

After extracting data, only 31 articles in the group studying pneumonia had any of the predictors that were present in at least 3 studies. The 31 articles were included in the meta-analysis. Eighteen other articles [10–27] met the initial inclusion criteria, but were later excluded from the meta-analysis: one was excluded because the results were presented with relative risk; all other were excluded because they did not report on predictors existing in 3 or more studies.

Neither UTI nor all-cause fever were possible to analyze because none of the predictors were described in at least 3 studies. Therefore, the results from these were instead presented descriptively in texts.

### Predictors of post-stroke pneumonia

The quality assessments of the 31 studies included in the meta-analysis are presented in Table 1. No article included met the criteria of low risk of bias, 17 studies were assessed to have average risk of bias and 14 as having a high risk of bias. Fourteen predictors were presented in 3 or more different studies and synthesized (Table 2). Age (for each year increase), male sex, NIHSS (by one point increase), dysphagia, nasogastric tube, diabetes, mechanical ventilation, smoking, chronic obstructive pulmonary disease (COPD) and atrial fibrillation were statistically significant predictors of post-stroke pneumonia. Previous stroke, dysphagia screening and hypertension were not statistically significantly associated with post-stroke pneumonia. I^2^s were generally high for all factors in the meta-analysis, indicating high level of heterogeneity.

**Table 1:** Studies included in pneumonia meta-analysis

Author	Quality assessment	Study design	Number of included participants
Almedia SR 2015 [40]	Average risk of bias.	Retrospective cohort.	159
Alsumrain M. 2013 [41]	Average risk of bias.	Retrospective cohort.	290
Brogan E. 2015 [32]	High risk of bias.	Retrospective cohort.	533
Brogan E. 2014 [42]	High risk of bias.	Retrospective cohort.	533
Bruening T. 2015 [43]	Average risk of bias	Prospective cohort.	538
Chen CM. 2012 [28]	High risk of bias.	Retrospective cohort.	341
Chumbler NR. 2010 [44]	Average risk of bias.	Retrospective cohort.	925
Colbert JF. 2016 [45]	Average risk of bias.	Retrospective cohort.	1225
Dziedzic T. 2006 [46]	Average risk of bias.	Retrospective cohort.	705
Gargano JW 2008 [47]	High risk of bias.	Prospective cohort.	2566
Hoffmann S. 2012 [48]	High risk of bias.	Retrospective cohort. Register.	Derivation cohort: 15,335 Validation cohort: 45,085
Hoffmeister L. 2013 [49]	Average risk of bias.	Retrospective cohort.	677
Hug A. 2009 [50]	High risk of bias.	Case-control study.	Case: 50 Control: 40
Ingeman A. 2011 [37]	Average risk of bias.	Retrospective cohort.	11,757
Ji R. 2013 [51]	High risk of bias.	Prospective cohort.	8820
Ji R. 2014 [52]	High risk of bias.	Prospective cohort.	4998
Kwon HM. 2006 [53]	Average risk of bias.	Prospective cohort.	286
Lakshminarayan K. 2010 [54]	Average risk of bias.	Retrospective cohort.	18,017
Li Y. 2014 [55]	Average risk of bias.	Prospective cohort.	1142
Liao CC. 2015 [56]	High risk of bias	Retrospective cohort.	211,256
Maeshima S. 2014 [57]	High risk of bias	Prospective cohort.	292
Marciniak C. 2009 [58]	High risk of bias.	Case-control.	Case: 36 Control: 36.
Masiero S. 2008 [23]	High risk of bias.	Prospective cohort.	67
Masrur S. 2013 [59]	Average risk of bias.	Retrospective cohort.	Analysis1: 304084 Analysis 2: 136452
Ribeiro PW. 2015 [60]	Average risk of bias	Prospective cohort.	70
Scheitz F. J. 2015 [61]	High risk of bias.	Prospective cohort.	481
Smith CJ. 2015 [62]	High risk of bias.	Secondary analysis. Prospective cohort.	11,551
Sui R. 2011 [63]	Average risk of bias.	Prospective cohort.	1435
Warnecke T. 2009 [64]	Average risk of bias.	Prospective cohort.	153
Yamamoto K. 2014 [65]	Average risk of bias.	Retrospective cohort.	133
Zhang X. 2012 [66]	Average risk of bias.	Prospective cohort.	106

**Table 2:** Predictors and associated ORs of post-stroke pneumonia

Predictor	Overall combined OR with *p*-value	I^2^ with p-value	Number of studies
Age [28, 39, 42–43, 45, 50–52, 54–57]	1.07 (1.04–1.11) *p* < 0.001	98.5% p < 0.001	12
Male [28, 32, 38, 44–45, 52–53, 55–56]	1.42 (1.17–1.74) *p* = 0,001	77.8% p < 0.001	9
NIHSS [28, 40, 42–33, 50–52, 55, 57]	1.07 (1.05–1.09) p < 0.001	82.4% * *P* < 0.001	9
Dysphagia [29, 34, 38, 42–45, 48, 51–52, 57]	3.53 (2.69–4.64) p < 0.001	86.6% p < 0.001	11
Nasogastric tube [29, 31, 33, 49, 54]	5.29 (3.01–9.32) p < 0.001	65.3% *p* = 0.01	5
Diabetes [35, 38–39, 47, 50, 52, 54, 56]	1.15 (1.08–1.23) p < 0.001	77.9% p < 0.001	8
Mechanical ventilation [29, 44, 49, 54]	4.65 (2.50–8.65) p < 0.001	61.8% *p* = 0.02	4
Previous stroke [32, 46, 50]	1.03 (0.96–1.10) *p* = 0.438	77.5% p < 0.001	3
Smoking [42–43, 50]	1.16 (1.08–1.26) p < 0.001	75.6% p < 0.001	3
COPD [23, 42–43]	4.48 (1.82–11.00) *p* = 0.001	81.1% p < 0.001	3
Atrial fibrillation [38, 42, 50]	1.37 (1.22–1.55) p < 0.001	93.7% p < 0.001	3
Dysphagia screening [39, 41, 50]	1.17 (0.95–1.43) *p* = 0.145	91.5% p < 0.001	3
Hypertension [36, 39, 50]	0.95 (0.87–1.04) *p* = 0.232	85.1% p < 0.001	3

### Predictors of UTI and all-cause fever

Eleven studies reporting predictors of UTI were eligible for meta-analysis, as presented in Table 3. However, since none of their predictors were described in at least 3 studies, no meta-analysis of predictors for UTI was possible. Statistically significant predictors in the individual studies were various laboratory analyses (procalcitonin, c-reactive protein, white blood cell count, monocytes count and copeptin), age, examination with computer tomography (CT)/magnetic resonance imaging (MRI), use of beta-blocker, female sex, previous pressure ulcer, mean rehabilitation ward stay, three variables pertaining to bladder dysfunction (post void residual volume > 50 ml or > 150 ml and incontinence on admission) and two variables connected to stroke severity (post-stroke modified Rankin Scale and lesion size ≥1.5 cm).

**Table 3:** Eligible studies presenting predictors of UTI

Author	Study design	Number of included participants	Significant predictors	Significant protective factors	Non-significant factors	Quality assessment
Fluri F. 2012 [11]	Prospective cohort.	383	In all models: Procalcitonin, c-reactive protein, white blood cell count, Monocytes. Model 1,3,4: Copeptin.	–	In all models: Body temperature. Model 2: Copeptin.	Average risk of bias.
Minnerup J. 2010 [67]	Prospective cohort.	594	–	Lesion size < 1,5 cm.	Lesion size 1,5–5,0 cm or 1/3 of MCA. Lesion size > 5 cm or > 1/3 of MCA.	Average risk of bias.
Stott DJ. 2009 [29]	Prospective cohort.	412	Urinary catheter, post-stroke modified Rankin Scale, age by decade,	–	–	Average risk of bias.
Dromerick AW. 2003 [30]	Retrospective cohort.	101	Beta-blocker, post void residual \volume > 150 ml,	–	Age > 65, Motor syndrome, Male, Anti-depressant,	High risk of bias.
Brogan E. 2014 [32]	Retrospective cohort.	533	Incontinence on admission.	–	–	High risk of bias.
Chen CM. 2012 [28]	Retrospective cohort.	341	Mean rehabilitation ward stay, post void residual volume > 50 ml,	–	Mean acute ward stay, ischemic stroke.	High risk of bias.
Ersoz M. 2007 [68]	Prospective cohort.	110	–	–	Urinary catheter.	Average risk of bias.
Ingeman A. 2010 [37]	Retrospective cohort; registry.	11,757	Examination with CT/MRI.	Early mobilization.	Early admission to stroke unit, antiplatelet therapy, anticoagulant therapy, assessment by physiotherapist, assessment by occupational therapist, assessment of nutritional risk, dysphagia screening,	High risk of bias.
Lee SY. 2016 [25]	Retrospective cohort; registry.	3002	Previous pressure ulcer.	–	–	High risk of bias.
Gargano JW. 2008 [47]	Retrospective cohort; registry.	2566	Female	–	–	High risk of bias.
Kwan J. 2004 [26]	Case-control	351	–	Integrated care pathway in acute stroke unit.	–	High risk of bias.

Only one article with all-cause fever as outcome was included (Table 4) after applying the exclusion criteria, and therefore no meta-analysis was possible. Three other articles were excluded because all patients were treated at intensive care units.

**Table 4:** Eligible studies presenting predictors for all-cause fever

Author	Study design	Number of included participants	Significant Predictors	Quality assessment
Muscari A. 2015 [38]	Retrospective cohort	536	Primary analysis, fever > 24 h after admission: Nasogastric tube, Atrial fibrillation, Total anterior circulation syndrome, Urinary catheter Secondary analysis fever < 24 h after admission: NIHSS, Hemorrhagic stroke, Atrial fibrillation, Total parenteral nutrition.	Average risk of bias.

## Discussion

Several factors in the meta-analyses were associated with post-stroke pneumonia, i.e. age, male sex (with corresponding negative association for female sex), NIHSS, dysphagia, nasogastric tube, diabetes, mechanical ventilation, smoking, COPD and atrial fibrillation. The articles studying UTI and all-cause fever were too few to synthesize. The main aim of the current study was to compile the existing evidence and compare the predictors of post-stroke pneumonia, UTI and all-cause fever. However, since only one meta-analysis was possible, we allow ourselves to make some tentative comparisons between our pneumonia meta-analysis and the studies on UTI and all-cause fever instead.

Some differences between the pneumonia meta-analysis and the eleven UTI articles call for special attention. The most obvious difference was that urinary catheters and other variables connected to bladder dysfunction were associated to UTI in several studies [28–30], but not to pneumonia, which reasonably reflects the well-known association between the use of urinary catheters and UTI [31]. In one of the included studies by Brogan et al., incontinence was a predictor of both UTI and pneumonia, possibly because incontinence may serve as a surrogate marker of stroke severity, high age or general frailty [32]. Further, UTI was in one study associated with female sex, mirroring the well-known over-representation of UTI in women [33]. In contrast, our meta-analysis revealed that males were 42% more prone to develop post-stroke pneumonia. This sex-difference may reflect an actual incidence disparity between males and females [34, 35], possibly driven by higher prevalence of current and past smoking in males in these age groups [36]. Another possible explanation is that the results may be biased by the well-known relative under-representation of UTI in males, which may prompt the clinician to suspect airway infections rather than UTI in males with post-stoke fever, while suspecting UTI in women. In addition to the study by Brogan et al. above [32], some of the included studies compared predictors of pneumonia versus UTI. Ingeman et al. [37] found no differences in predicting factors between UTI and pneumonia, while Chen et al. [28] found that ischemic stroke as well as post-void residual volume increased the risk for UTI, while these factors were non-significant for pneumonia.

Comparisons become even more precarious when it comes to all-cause fever, since only one study was found eligible. Further, the patients suffering from all-cause fever reasonably constitute a mix of several different etiologies. Indeed, Muscari et al. [38] (Table 4) found that the strongest predictor of all-cause fever was use of nasogastric tube, which mechanistically ought to be associated with pneumonia, in line with our meta-analysis. Also, they found that urinary catheter was a risk factor for fever, reasonably because UTI caused part of the fevers [38]. An interesting detail was that fever within 24 h after stroke was not significantly related to these infection-associated variables, but rather to variables more connected to stroke severity (NIHSS, hemorrhagic stroke, atrial fibrillation and total parenteral nutrition), which might indicate that early fever to a larger extent is non-infectious [38].

Regarding diabetes, COPD, atrial fibrillation, nasogastric tube and mechanical ventilation, the results of this study corroborates the results of a previous meta-analysis of risk factors for post-stroke lung infection by Yuan et al. [39]. Yuan et al. also showed increased risk for post-stroke lung infections for the variables Age above 65 years, NIHSS 5–15 and NIHSS > 15 [39], which was also confirmed by the current results, even if we analyzed age and NIHSS as continuous variables. In contrast to the current study, Yuan et al. found that male sex and smoking did not significantly increase the risk for post-stroke lung infection, while hypertension and previous stroke did [39]. The study by Yuan et al. adopted wider inclusion criteria, for example not demanding the results to be expressed as odds ratio, which partly could explain these differences [39].

### Limitations

The meta-analysis of post-stroke pneumonia showed high rates of heterogeneity, which could suggest that the included studies are not fully comparable. This could at least partly be explained by that the studies included different independent variables in their regression models. Moreover, a majority of the pneumonia articles studied pneumonia or lung-infection during hospitalization, while remaining articles studied pneumonia in a longer time perspective (within 4 days to 30 days post-stroke). Moreover, the studies were included regardless of how they defined pneumonia. Hence, the heterogeneity might be caused by the differences in defining pneumonia in the included studies. To compensate for some of the heterogeneity, only studies performing multiple logistic regressions were included. A further note of caution is that the bias risk of the included studies was generally average or high. Moreover, only one investigator assessed the articles and extracted data, which may limit the reliability of the results. In addition to this only one database was searched, which leaves a possibility that not all relevant studies were included.

## Conclusions

We conclude that post-stroke pneumonia is predicted by age, male sex, NIHSS, dysphagia, nasogastric tube, diabetes, mechanical ventilation, smoking, COPD and atrial fibrillation. An opposite relation to sex may exist for UTI, which seems to be more common in women. The lack of studies simultaneously studying a wide range of predictors for UTI or all-cause fever calls for future research in this area. The importance of new research would be to improve our understanding of fever complications to facilitate greater vigilance, monitoring, prevention, diagnosis and treatment..

## Abbreviations

CI: Confidence interval
COPD: Chronic obstructive pulmonary disease
CT: Computer tomography
MRI: Magnetic resonance imaging
NIHSS: National Institute of Health stroke scale
OR: Odds ratio
UTI: Urinary tract infection
